# Factors Influencing the Therapeutic Efficacy of the PSMA Targeting Radioligand ^212^Pb-NG001

**DOI:** 10.3390/cancers14112784

**Published:** 2022-06-03

**Authors:** Vilde Yuli Stenberg, Anna Julie Kjøl Tornes, Hogne Røed Nilsen, Mona-Elisabeth Revheim, Øyvind Sverre Bruland, Roy Hartvig Larsen, Asta Juzeniene

**Affiliations:** 1 Department of Radiation Biology, Institute for Cancer Research, The Norwegian Radium Hospital, Oslo University Hospital, 0379 Oslo, Norway; anna.julie.kjol.tornes@rr-research.no (A.J.K.T.); asta.juzeniene@rr-research.no (A.J.); 2 Nucligen AS, 0379 Oslo, Norway; sciencons@gmail.com; 3 Institute of Clinical Medicine, University of Oslo, 0318 Oslo, Norway; monar@ous-hf.no (M.-E.R.); osb@ous-hf.no (Ø.S.B.); 4 Department of Pathology, Rikshospitalet, Oslo University Hospital, 0372 Oslo, Norway; hogne.roed.nilsen@rr-research.no; 5 Division of Radiology and Nuclear Medicine, Oslo University Hospital, 0379 Oslo, Norway; 6 Department of Oncology, The Norwegian Radium Hospital, Oslo University Hospital, 0379 Oslo, Norway

**Keywords:** prostate-specific membrane antigen, metastatic castration-resistant prostate cancer, targeted alpha therapy, NG001, ^212^Pb

## Abstract

**Simple Summary:**

Prostate-specific membrane antigen (PSMA) is a protein overexpressed in metastatic castration-resistant prostate cancer and a promising target for targeted radionuclide therapy. PSMA-targeted alpha therapy is of growing interest due to the high-emission energy and short range of alpha particles, resulting in a prominent cytotoxic potency. This study assesses the influence of various factors on the in vitro and in vivo therapeutic efficacy of the alpha particle generating PSMA-targeting radioligand ^212^Pb-NG001.

**Abstract:**

This study aimed to determine the influence of cellular PSMA expression, radioligand binding and internalization, and repeated administrations on the therapeutic effects of the PSMA-targeting radioligand ^212^Pb-NG001. Cellular binding and internalization, cytotoxicity, biodistribution, and the therapeutic efficacy of ^212^Pb-NG001 were investigated in two human prostate cancer cell lines with different PSMA levels: C4-2 (PSMA+) and PC-3 PIP (PSMA+++). Despite 10-fold higher PSMA expression on PC-3 PIP cells, cytotoxicity and therapeutic efficacy of the radioligand was only 1.8-fold better than for the C4-2 model, possibly explained by lower cellular internalization and less blood-rich stroma in PC-3 PIP xenografts. Mice bearing subcutaneous PC-3 PIP xenografts were treated with 0.2, 0.4, and 0.8 MBq of ^212^Pb-NG001 that resulted in therapeutic indexes of 2.7, 3.0, and 3.5, respectively. A significant increase in treatment response was observed in mice that received repeated injections compared to the corresponding single dose (therapeutic indexes of 3.6 for 2 × 0.2 MBq and 4.4 for 2 × 0.4 MBq). The results indicate that ^212^Pb-NG001 can induce therapeutic effects at clinically transferrable doses, both in the C4-2 model that resembles solid tumors and micrometastases with natural PSMA expression and in the PC-3 PIP model that mimics poorly vascularized metastases.

## 1. Introduction

Prostate-specific membrane antigen (PSMA) is a cell surface glycoprotein overexpressed in metastatic castration-resistant prostate cancer (mCRPC) [1,2,3]. The rapid constitutive internalization of bound PSMA radioligands makes it an ideal antigen for molecular targeted radionuclide therapy (TRT) [4,5]. Recently published results of the phase III VISION trial (NCT03511664) demonstrated significantly improved overall survival and radiographic progression-free survival in patients treated with the beta-emitting ^177^Lu-PSMA-617 [6]. However, 30–40% of patients do not respond to or are not suitable for this therapy due to diffuse bone marrow infiltration [7,8,9,10]. It has been demonstrated that treatment may be more effective when PSMA-617 is labeled with the alpha emitter ^225^Ac instead of ^177^Lu [11,12,13,14]. Consequently, PSMA-targeted alpha therapy (TAT) has gained increased interest for the treatment of mCRPC.

Several novel PSMA-targeting agents (small molecule ligands, peptides, antibodies, and aptamers) labeled with ^149^Tb (t_1/2_ ≈ 4.1 h), ^211^At (t_1/2_ ≈ 7.2 h), ^212^Pb (t_1/2_ ≈ 10.6 h), ^225^Ac (t_1/2_ ≈ 9.9 d), and ^227^Th (t_1/2_ ≈ 18.7 d) are currently being evaluated in preclinical studies for TAT of mCRPC [14,15,16,17,18,19,20,21,22,23]. However, the direct comparison of published data from various research groups is challenging because a range of prostate cancer cell lines, mouse strains, and molar amounts and activities of the radioligands have been used. The human prostate cancer PC-3 PIP, LNCaP, and C4-2 cell lines are the most often used in preclinical evaluations of PSMA-targeting radioligands [15,17,18,24,25,26,27,28,29,30]. LNCaP and C4-2 display 1.4–5.9 × 10^5^ PSMA sites per cell [23,29,31,32,33,34,35,36], whereas PC-3 PIP has been genetically engineered to stably express PSMA levels 10–20 times higher (4.9 × 10^6^ sites per cell) [35]. It has been suggested that prostatic subcutaneous tumors with higher PSMA expression levels respond better to TRT because higher radioligand binding may increase tumor accumulation and DNA damage [36]. Current et al. demonstrated that PC-3 PIP xenografts with higher ratios of PSMA-positive to PSMA-negative cells resulted in higher tumor uptake and therapeutic efficacy of ^177^Lu- and ^225^Ac-labeled PSMA-617 [36]. However, the use of the same cell line in variable ratios does not consider other characteristics that may differ in various tumors, e.g., cellular binding and internalization, growth pattern of the cell line in vivo, and tumor vascularization. Tschan et al. showed that the cell type plays an important role in evaluating radioligands in vitro and in vivo [25].

The subcutaneous tumor microenvironment (blood vessel density and hypoxic regions) plays an important role for PSMA radioligand delivery and dose distribution in the tumor [37,38]. Interestingly, PSMA is a regulator of new blood vessel formation (i.e., angiogenesis) [39,40]. Ngen et al. detected higher vascular densities and blood vessels of larger diameters at the tumor peripheries compared to the tumor centers of PC-3 PIP tumors, and found that the intratumoral vascular density and distribution pattern influenced the delivery and distribution of their PSMA-targeted nanoparticles [41]. In addition, rapid tumor growth may influence radioligand uptake because vascular density tends to decrease as the tumor grows, leading to zones of hypoxia and eventually necrosis as tumors “outgrow” their blood supply [42].

Another aspect that makes direct comparisons of different radiopharmaceuticals challenging is the use of a range of molar amounts and activities of radioligands. This can lead to variable degrees of receptor saturation in PSMA-expressing tissues, thereby influencing uptake [25,43,44,45]. Some studies report increased tumor and kidney uptake at increasing effective molar activities (MBq/nmol of ligand) [25,43,44,46,47], whereas others observe no such correlation [45,48,49]. Thus, it is challenging to interpret how the molar activity of radioligands may impact tumor and kidney accumulation. A strategy to minimize damage to healthy tissue while maintaining the maximized cumulative uptake in the tumor have been successfully demonstrated through multiple injections of alpha emitting radionuclides (NCT04576871, NCT04506567) [48,50,51,52].

Finally, the radiation sensitivity of the mouse model used in various studies must be taken into consideration. Inferior repair capacity against DNA damage in radiosensitive strains, such as severe combined immunodeficient (SCID) and non-obese diabetic (NOD)-SCID gamma (NSG) mice, is thought to result in prolonged tumor growth delay after radiation exposure compared to less radiosensitive strains, such as NOD rag gamma and athymic nude mice [53,54,55]. To our knowledge, direct comparisons using identical treatments of radioligand in different murine models have not yet been performed.

In the present study, the small molecule PSMA ligand NG001 [23], labelled with ^212^Pb, was studied in PC-3 PIP and C4-2 prostate cancer models in vitro and in vivo to better understand how cellular PSMA expression, binding, and internalization influence biodistribution and the therapeutic efficacy of the radioligand. The effect of repeated administrations of the radioligand on therapeutic efficacy was also investigated. In addition, the anti-tumor activity of ^212^Pb-NG001 was compared in mouse models with different radiation sensitivities.

## 2. Materials and Methods

### 2.1. Preparation of ^212^Pb and Activity Measurements

Lead-212 was produced from ^224^Ra via ^220^Rn emanation by using a simplified single-chamber generator system [56]. The ^224^Ra was extracted from ^228^Th (Eckert & Ziegler, Braunschweig, Germany or Oak Ridge National Laboratory, Oak Ridge, TN, USA) by using column-based generators as described by Westrøm et al. [57]. The ^224^Ra/^212^Pb generator consisted of a glass flask turned upside down and a removable cap that contained the ^224^Ra solution (in 0.1 M HCl) absorbed onto a porous holding material. During ^224^Ra decay, ^220^Rn emanated from the holding material and caused the absorption of ^212^Pb onto the interior surfaces of the flask. After 1–2 days, the flask was carefully removed from the source cap and rinsed with 0.1 M HCl to dissolve the ^212^Pb deposits. The extracted ^212^Pb was of high purity with ^224^Ra breakthrough below 0.1% [58,59,60].

To determine ^212^Pb activity, radioactive samples were measured on a Hidex Automatic Gamma counter (Hidex Oy, Turku, Finland) with the 60–110 keV counting window and in a Capintec CRC-25R radioisotope dose calibrator (Capintec Inc., Ramsey, NJ, USA) with calibration number 667 for ^212^Pb in equilibrium with progenies [58,61]. The 520–640 keV window was used to estimate ^212^Bi activity indirectly from the highly abundant ^208^Tl gamma radiation (30.6% abundance) [61].

### 2.2. Radiolabelling of NG001 with ^212^Pb

The PSMA ligand NG001, supplied as the dried trifluoroacetic acid salt with purity of ≥98% (MedKoo Biosciences, Morrisville, NC, USA) [23], was dissolved in 0.5 M ammonium acetate (NH_4_OAc) in 0.1 M HCl. Lead-212 was extracted in 0.1 M HCl from the generator and 0.5 M sodium acetate was added, which adjusted the pH to 5–6. NG001 was added to the ^212^Pb solution, typically 1–3 nmol in 0.2–0.5 mL, and incubated for 25 min on a Thermomixer (Eppendorf, Hamburg, Germany) at 37–60 °C and 450–650 rpm. The radiochemical purity of the radioligand was evaluated by thin layer chromatography by using instant thin layer chromatography strips (Tec-control, model #150-772, Biodex Medical Systems, Shirley, NY, USA). Radioligands with radiochemical purity >95% were used for experiments.

### 2.3. Cell Lines

The human prostate cancer cell lines C4-2 (0.1–0.4 × 10^6^ PSMA binding sites per cell) and PC-3 PIP (4.9 × 10^6^ PSMA binding sites per cell) were used in the studies [15,23,29,32,34,35,36]. In addition, the PC-3 Flu cell line (2.5 × 10^3^ PSMA binding sites per cell) was used as a negative control in the clonogenic assay [35]. PC-3 Flu (PSMA-) and PC-3 PIP (PSMA+++) cells were provided by Dr. Martin Pomper (John Hopkins University School of Medicine, Baltimore, MD, USA), and C4-2 (PSMA+) cells were obtained from ATCC (ATCC^®^ CRL-3314™, Manassas, VA, USA). Cells were grown in RPMI 1640 medium (Sigma-Aldrich Norway AS, Oslo, Norway) supplemented with 10% heat-inactivated fetal bovine serum (FBS, GE Healthcare Life Sciences, Chicago, IL, USA), 100 units/mL penicillin, and 100 µg/mL streptomycin (Sigma-Aldrich Norway AS, Oslo, Norway). In addition, the PC-3 PIP cell line was supplemented with puromycin (2 µg/mL) to maintain PSMA expression [62]. Cell cultures were grown at 37 °C in a humid atmosphere with 5% CO_2_.

### 2.4. Binding and Internalization of ^212^Pb-NG001 in C4-2 and PC-3 PIP Cells

Radioligand binding was measured in PC-3 PIP and C4-2 cells by incubating 10^6^ cells in 0.2 mL of PBS supplemented with 0.5% bovine serum albumin (BSA, Sigma-Aldrich Norway AS, Oslo, Norway) with eight different radioligand concentrations (3–150 nM) for 1 h at 37 °C and 100 min^−1^. Nonspecific binding was estimated by blocking cells with an excess of unlabeled ligand, 12 µM, for 15 min before the addition of radioligand (199 kBq/nmol). Activities of the samples were measured in a gamma counter before (added activity) and after cells had been washed three times with 0.5% BSA in PBS (cell-bound activity). For the three lowest concentrations, internalization was determined by incubating samples with 1 mL of 50 mM glycine stripping buffer in 150 mM NaCl (pH 2.8) for 10 min at room temperature. The cells were washed three times with PBS containing 0.5% BSA to remove surface bound radioactivity and were then measured in a gamma counter (internalized activity). Specific cell-bound activity was calculated as the percentage of added activity, whereas internalized activity was calculated as the percentage of specific cell-bound activity.

Retention of cell-bound ^212^Pb and ^212^Bi activities was determined as above but with 5 × 10^6^ cells per sample. After cell-bound activity was measured, the cells were further incubated in 1 mL of fresh medium for 1, 2, 4, and 24 h at 37 °C and 100 min^−1^. The cells were vortexed and centrifuged and the retained cell-bound activity was measured in a gamma counter. All binding and internalization experiments were performed in duplicates, 2–5 independent times.

### 2.5. Clonogenic Assay

The radiotoxicity of X-ray radiation and ^212^Pb-NG001 in PC-3 Flu, C4-2, and PC-3 PIP cells was studied by clonogenic assay where each treatment condition was performed in triplicates and repeated 2–3 independent times for each cell line. The toxicity of X-ray radiation was assessed by collecting 0.5 × 10^6^ cells in 2 mL growth medium following irradiation at seven different activities (0.5–5 Gy). After irradiation, the cells were resuspended in media and 1000–50,000 cells were seeded in 25 cm^2^ flasks (three flasks per group). The toxicity of ^212^Pb-NG001 was assessed by incubating 0.5 × 10^6^ cells with seven different activity concentrations (effective molar activity of 404 kBq/nmol) of the radioligand (5–150 kBq/mL) in 0.2 mL of growth medium for 1 h at 37 °C and 100 min^−1^. After incubation, the cells were washed with fresh medium and 1000–10,000 cells were seeded in 25 cm^2^ flasks (three flasks per group). The flasks were incubated at 37 °C and 5% CO_2_ for 7–14 days until colonies were large enough for staining (minimum 50 cells per colony). The colonies were fixated with ethanol and stained with 0.4% methylene blue (Thermo Fischer Scientific, Waltham, MA, USA). The colonies were counted, and plating efficiencies and survival fractions were calculated.

To assess binding and internalization at different activity concentrations, the remaining cells after seeding in flasks were transferred to glass tubes and washed with 0.5% BSA in PBS before the cell-bound activity was measured in a gamma counter. Subsequently, cells were incubated with 1 mL of 50 mM glycine stripping buffer (in 150 mM NaCl, pH 2.8) for 10 min, centrifuged, and internalized activities were measured. The clearance of internalized radioactivity was assumed to be only due to radioactive decay. PC-3 Flu cells were used as a negative control. The total cell-bound or internalized activity per cell (Bq/cell) and number of ^212^Pb atoms per cell were calculated.

### 2.6. Animals and Tumor Xenografts

In vivo studies were performed with 108 male Hsd: Athymic Nude-Foxn1^nu^ and 9 male NOD.Cg-Prkdcscid Il2rgtm1Wjl/SzJ (NSG) mice bred at the Department of Comparative Medicine at the Norwegian Radium Hospital (Oslo University Hospital, Oslo, Norway). The studies were approved by the Institutional Committee on Research Animal Care and the Norwegian Food Safety Authority (Brumunddal, Norway, approval: FOTS ID 22197). All studies complied with the Interdisciplinary Principles and Guidelines for the Use of Animals in Research, Marketing, and Education (New York Academy of Sciences, New York, NY, USA) and the EU Directive 2010/63/EU for animal experiments. The cages (up to five mice per cage) were maintained under specific pathogen-free conditions with constant temperature (24 °C) and humidity (60%), and the mice had access to food and water ad libitum. The mice were 5–8 weeks in age, weighing between 25–35 g at the start of the study.

Mice were inoculated subcutaneously in both flanks with 5 × 10^6^ PC-3 Flu cells/flank, 10 × 10^6^ C4-2 cells/flank or 5 × 10^6^ PC-3 PIP cells/flank in RPMI1640 medium without supplements mixed 1:1 with Matrigel Matrix (Corning Inc., Corning, NY, USA), in a total volume of 200 µL. The tumors were allowed to grow to a volume between 150 and 1000 mm^3^ for the biodistribution studies. For the histology and therapy studies, tumor volumes were between 40 and 480 mm^3^ in PC-3 PIP xenografts, and 15 and 400 mm^3^ in C4-2 xenografts.

### 2.7. Immunohistochemical (IHC) and Hematoxylin and Eosin (H&E) Staining

PC-3 Flu, C4-2, and PC-3 PIP xenografts and kidneys from non-treated mice were collected for histology and fixed in 4% formaldehyde in phosphate buffer (pH~7). Fixed tissue samples were paraffin-embedded and cut in 4-µm sections by using a microtome (2 slides per sample). For IHC PSMA expression, one set of the samples was deparaffinized, re-hydrated, and subjected to heat-induced antigen retrieval in a water bath for 20 min at 95 °C by using Dako Target Retrieval Solution pH9 (S2367, Agilent Technologies, Santa Clara, CA, USA). Endogenous peroxidase was quenched by using Dako Peroxidase-Blocking Solution (S2023, Agilent Technologies, Santa Clara, CA, USA) before tissue sections were probed with a rabbit monoclonal antibody to PSMA (SP29, 1:100 dilution, Abcam, Cambridge, UK) for 60 min at room temperature. Bound antibodies were detected by using Rabbit-on-Rodent HRP-Polymer (RMR622, Biocare Medical, Pacheco, CA, USA) and Dako Liquid DAB+ Substrate Chromogen System (K3468, Agilent Technologies, Santa Clara, CA, USA). Finally, sections were counterstained in hematoxylin, dehydrated, and mounted in Pertex. An isotype control antibody (#3900, Cell Signaling, Danvers, MA, USA) was included in the IHC staining. The other set of samples was stained with H&E (Dako, Agilent Technologies, Santa Clara, CA, USA). The stained samples were examined by using an automatic slide scanner (VS200, Olympus, Tokyo, Japan) and images were taken by using VS200 DESKTOP ASW 3.2 software (Olympus, Tokyo, Japan).

### 2.8. Biodistribution of ^212^Pb-NG001 in Mice with PC-3 PIP Xenografts

Mice were injected intravenously via the tail vein with 10–60 kBq (0.05–0.90 nmol) of ^212^Pb-NG001. At predefined time points after injection (1, 2, 4, and 24 h), blood was collected by cardiac puncture while the mice were under gas anesthesia (~3.5% Sevoflurane in oxygen at 0.5 L/min; Baxter, IL, USA). Mice were euthanized by cervical dislocation and different organs were harvested. The weight and activity of each tissue sample was measured in a gamma counter and the decay-corrected percentage of injected dose per gram tissue (%ID/g) was calculated from injection standards. The absorbed radiation doses from ^212^Pb-NG001 in tumors were calculated by using the biodistribution data, normalized to an injection of 0.4 MBq per mouse. The area under the specific activity (Bq/g) versus time curves were multiplied with 7.9 MeV, which corresponds to the alpha energy from the decay of ^212^Pb [15]. Beyond the last time point, clearance of radioactivity was assumed to be only due to radioactive decay.

### 2.9. Therapeutic Effect of ^212^Pb-NG001 in Mice with PC-3 PIP and C4-2 Xenografts

The therapeutic effect of radioligands was studied in four independent studies. Mice with PC-3 PIP xenografts were injected intravenously via the tail vein either with a single administration of 0.20, 0.30, 0.35, or 0.75 MBq of ^212^Pb-NG001 (0.14–0.61 nmol), or with two administrations of 0.11 (total dose of 0.22 MBq), 0.20 (total dose of 0.40 MBq), or 0.38 (total dose of 0.76 MBq) MBq of ^212^Pb-NG001 (total of 0.13–0.60 nmol), injected 3 or 14 days apart. Mice with C4-2 xenografts were injected intravenously via the tail vein with a single administration of 0.80 or 0.85 MBq of ^212^Pb-NG001 (0.28–0.34 nmol). Control mice received 100 µL of 0.9% NaCl. Mice were monitored two to three times per week for changes in body weight and tumor size, and for any signs for termination criteria: weight loss of >20% from initial body weight, rapid weight loss of >10% within 2 days, tumors exceeded 20 mm in any direction, ulcerated or interfered with normal behavior, or any signs of severe sickness or discomfort. Blood was collected from the saphenous vein and complete blood counts were obtained by using the hematology analyzer Hycel Hycount 3N (Hycel Medical, Schwechat, Austria). Blood was also drawn by cardiac puncture under gas anesthesia, and serum was collected and analyzed. Glutamic oxaloacetic transaminase, glutamic pyruvic transaminase, alkaline phosphatase, bilirubin, urea, and creatinine were measured on a Reflotron Plus (Roche Diagnostics AS, Oslo, Norway). Selected organs (liver, spleen, kidneys, and tumor) were harvested and weighed.

### 2.10. Statistics

Statistical analyses were performed by using SigmaPlot 14.5 software (Systat Software, Inc., San Jose, CA, USA). Groups were compared by using a one-way ANOVA with multiple comparisons. Low numbers of samples (<5) were analyzed with non-parametric tests; parametric tests were chosen after testing for normality in data with *n* ≥ 5. Normality was assumed after a Shapiro–Wilk normality test. The survival of the mice was analyzed with Kaplan–Meier curves and a log-rank test. A *p*-value of < 0.05 was considered statistically significant.

## 3. Results

### 3.1. Cell Binding, Internalization, and Cytotoxicity of ^212^Pb-NG001 in C4-2 and PC-3 PIP Cells

**Figure 1:**
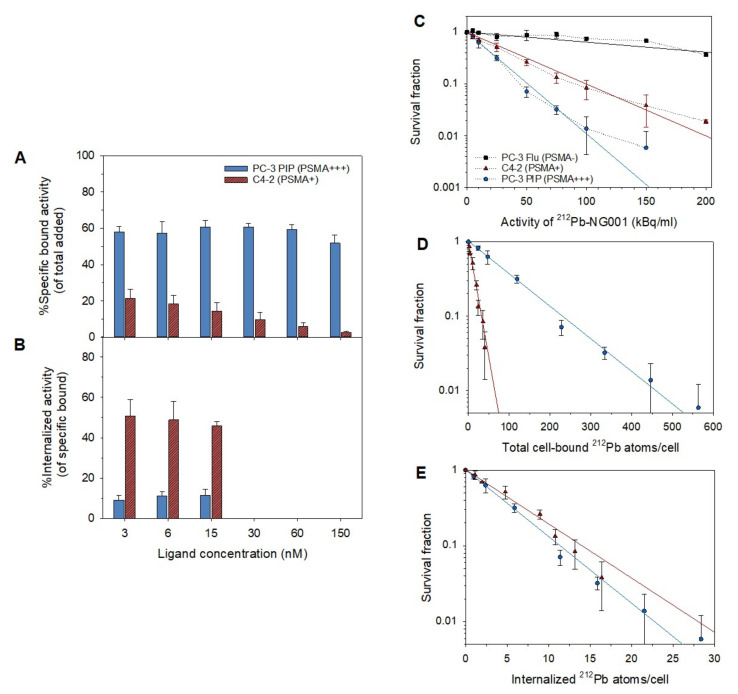
(**A**) Specific bound (% of total added activity) and (**B**) internalized activity (% of specific bound activity) of ^212^Pb-NG001 at variable ligand concentrations in C4-2 and PC-3 PIP cells, *n* = 3–5. (**C**) Survival fraction of PC-3 Flu, C4-2, and PC-3 PIP cells after 1 h treatment with ^212^Pb-NG001 (mean ± SD, *n* = 2–3) compared to untreated control cells. Survival fraction of C4-2 and PC-3 PIP cells at variable number of (**D**) bound and (**E**) internalized ^212^Pb atoms/cell. The cell survival curves (**C**) were fitted by using SigmaPlot 14.5 software by the single-hit model (solid line, equation: SF = exp(−A/A_0_)), where SF is survival fraction, A is the activity (kBq/mL), and A_0_ is the activity to reduce survival by 67%; dotted line, experimental data. The cell survival data (**D**,**E**) were fitted with single exponential functions.

The cell binding and internalization of ^212^Pb-NG001 were tested at variable ligand concentrations in PC-3 PIP (PSMA+++) and C4-2 (PSMA+) cells. The percentage of added activity bound specifically to cells was reduced at increasing ligand concentrations for C4-2 cells but not for PC-3 PIP cells (Figure 1A). In PC-3 PIP cells, the specific binding of ^212^Pb-NG001 was 59 ± 4% at ligand concentrations of 3–60 nM, whereas a 7% decrease was observed at 150 nM (52 ± 5%). Radioligand binding to C4-2 cells was 2.7 times lower than to PC-3 PIP cells at 3 nM (22 ± 5%) and was gradually reduced at increasing ligand concentrations (10 ± 4% at 30 nM and 3 ± 1% at 150 nM). In contrast, the internalized fraction was 4.8 times lower for PC-3 PIP cells (10 ± 3% of specific bound radioligand) than for C4-2 cells (48 ± 7% of specific bound) at 3, 6, and 15 nM (Figure 1B). The absolute amount of internalized radioligand was, thus, in a similar range for both cell lines (7 ± 2% of total added activity). The majority of the specific cell bound ^212^Pb-NG001 was retained over 24 h for both cell lines (Figure S1).

C4-2 and PC-3 PIP cells demonstrated similar radiosensitivity to X-ray radiation in a clonogenic assay (Figure S2 and Table S1). The dose required to reduce the fraction of surviving C4-2 and PC-3 PIP cells by 37% were 1.1 ± 0.1 and 1.2 ± 0.1 Gy, respectively. Survival of PC-3 Flu, C4-2, and PC-3 PIP cells showed dose-dependent killing at activity concentrations of 5–150 kBq/mL of ^212^Pb-NG001 (Figure 1C). The areas under the survival curves were obtained for PC-3 Flu, C4-2, and PC-3 PIP by fitting the curves to a single-target model. A 3.2-fold (*p* < 0.001) and a 5.4-fold (*p* < 0.001) decrease in survival was observed for C4-2 and PC-3 PIP, respectively, compared to PC-3 Flu. The PC-3 PIP cells required a higher number of bound ^212^Pb atoms/cell than C4-2 cells to yield a comparable clonogenic survival (70 versus 10 ^212^Pb atoms to obtain 50% survival, A50 of 15 and 29 kBq/mL, respectively; Figure 1D and Table S2), whereas the required number of internalized ^212^Pb atoms/cell was similar for both cell lines (3–5 ^212^Pb atoms at A50; Figure 1E and Table S2).

Total bound activity of ^212^Pb-NG001 per cell was 10–12-fold higher in PC-3 PIP than in C4-2 cells at all studied concentrations (Figure S3). Internalized activity per cell was similar for both cell lines at 5 and 10 kBq/mL, whereas it was 1.2–1.7 times higher in PC-3 PIP cells compared to C4-2 cells at higher activities of ^212^Pb (Figure S3).

### 3.2. Biodistribution of ^212^Pb-NG001 in Mice with PC-3 PIP and C4-2 Xenografts

**Figure 2:**
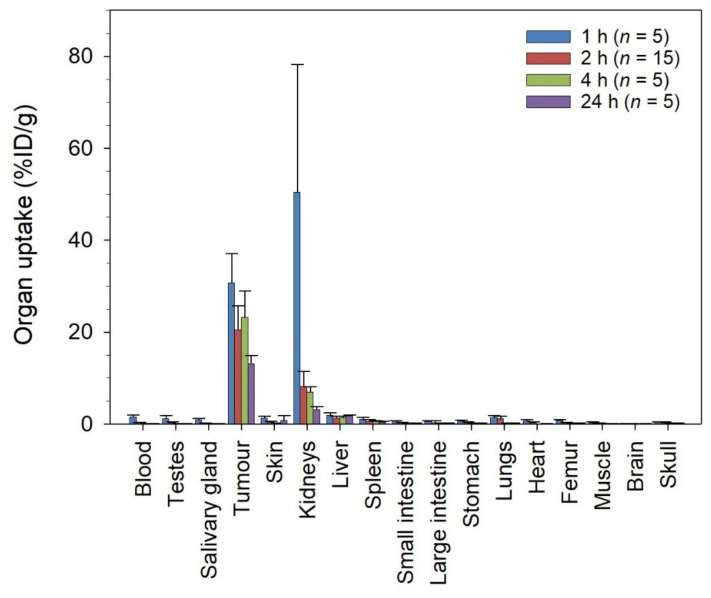
Uptake (percent of injected dose per gram of tissue, %ID/g) of ^212^Pb-NG001 in nude mice bearing human prostate PC-3 PIP xenografts at various time points after administration. *n*, number of mice per group.

The ^212^Pb-NG001 accumulated fast in PC-3 PIP tumors (30.7 ± 6.4%ID/g at 1 h; Figure 2) and showed long retention (>13%ID/g at 24 h). The radioligand was excreted mainly via the renal pathway, yielding high initial levels in urine and the kidneys (50 ± 28%ID/g) at 1 h post injection. However, radioactivity levels in kidneys were reduced more than 7-fold within 4 h and was below 3.5%ID/g at 24 h post injection. The uptake in other non-targeted tissues, including liver, spleen, intestines, salivary glands, and femur, was low with activity values below 1.5%ID/g at all studied time points. The uptake of ^212^Pb-NG001 in tumors and kidneys was similar as in a C4-2 xenograft model (Table S3) [16]. The only significant differences were higher tumor uptake in PC-3 PIP tumors at 4 h (23.2 ± 5.7 vs. 13.6 ± 2.1%ID/g, *p* = 0.007) and lower kidney uptake at 2 h post injection (8.2 ± 3.4 vs. 21.0 ± 10.3%ID/g, *p* = 0.001). The absorbed tumor radiation dose of ^212^Pb-NG001 was determined to be 3.1 Gy for the C4-2 tumors and 4.4 Gy for the PC-3 PIP tumors (Figure S4).

No significant correlation was found between the effective molar activity of ^212^Pb-NG001 (MBq/nmol of NG001) and radioligand uptake in PC-3 PIP tumors and kidneys at 2 h post injection (Figure S5). A trend of increased radioligand uptake in kidneys of smaller size was detected in PC-3 PIP-bearing mice and no significant difference between uptake and tumors of varying size was observed (Figure S5).

### 3.3. Therapeutic Effect of ^212^Pb-NG001 in Mice with PC-3 PIP Xenografts

**Figure 3:**
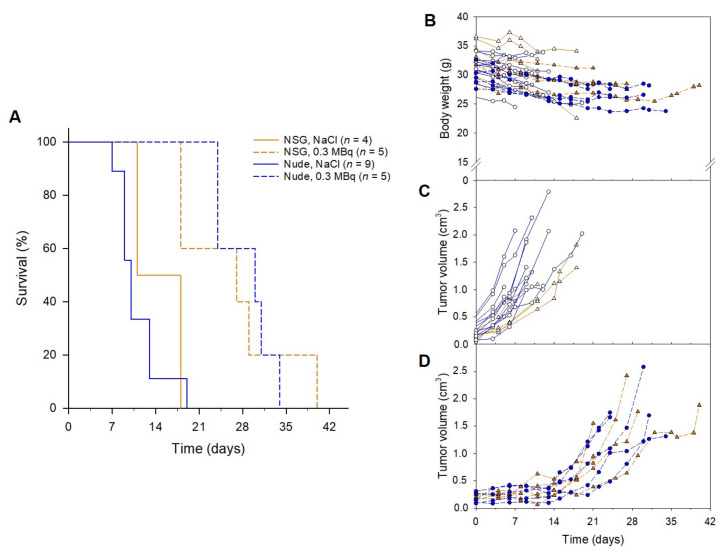
(**A**) Survival, (**B**) body weight and (**C**,**D**) tumor growth of NSG and athymic nude mice bearing human prostate PC-3 PIP tumors treated with (**C**) saline or (**D**) 0.3 MBq of ^212^Pb-NG001. Survival was estimated by Kaplan–Meier survival analysis followed by log-rank test by pairwise comparisons. *n*, number of mice per group.

The therapeutic effect of ^212^Pb-NG001 administered in a single dose was assessed in athymic nude and NSG mice bearing PC-3 PIP tumors. The median survival in the control groups were 10 and 14.5 days for nude and NSG mice, respectively (Figure 3). Tumor growth delay was observed in both mouse models administered 0.3 MBq of ^212^Pb-NG001, resulting in significantly improved median survivals of 30 days for nude and 27 days for NSG mice (*p* < 0.05 for treatment versus control groups; Figure 3). All mice lost 5–10% of body weight (Figure 3B). The lack of significant differences between the control groups (*p* > 0.05) and the treatment groups (*p* > 0.05) indicates that the therapeutic effect was independent of the mouse model. Thus, athymic nude mice were used for the rest of the animal studies.

**Figure 4:**
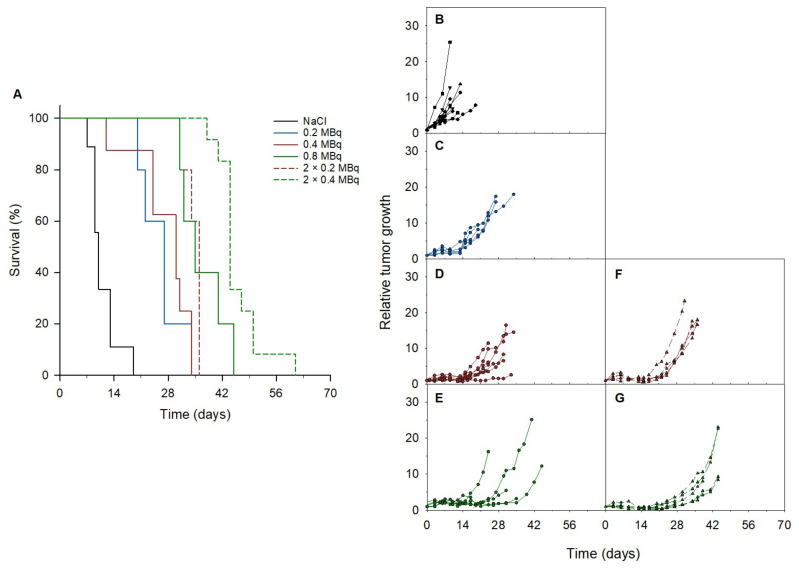
(**A**) Survival and (**B**–**G**) relative tumor growth of PC-3 PIP xenografts treated with (**B**) saline (*n* = 9), (**C**) 0.2 (*n* = 5), (**D**) 0.4 (*n* = 8), (**E**) 0.8 (*n* = 5), (**F**) 2 × 0.2 (*n* = 5) or (**G**) 2 × 0.4 (*n* = 12) MBq of ^212^Pb-NG001. Survival was estimated by Kaplan–Meier survival analysis followed by a log-rank test with multiple pairwise comparisons (Holm–Sidak). Each line in (**B**–**G**) represents the largest tumor from each mouse.

An improved median survival and tumor growth delay were observed in all treatment groups compared to the control group in athymic nude mice bearing PC-3 PIP tumors (Figure 4 and Table 1). Increasing doses of 0.2 MBq, 0.4 MBq, and 0.8 MBq of ^212^Pb-NG001 resulted in delayed tumor growth and extended survival. A significant increase in treatment response was detected in mice that received repeated doses compared to the corresponding single dose (Table 1 and Table S4). A therapeutic index (TI) of 3.5 was achieved in mice treated with 2 × 0.2 MBq of ^212^Pb-NG001 compared to a TI of 3.0 for mice treated with 0.4 MBq (Table 1). The highest TI was obtained in mice treated with 2 × 0.4 MBq compared to those treated with 0.8 MBq of ^212^Pb-NG001 (TI of 4.4 vs. 3.5; Table 1), regardless of the time of the second injection (3 or 14 days; Figure S6). It is noteworthy that the treatment of 0.8 MBq had a similar TI as 2 × 0.2 MBq, indicating a significant benefit of repeated injections. The TI of ^212^Pb-NG001 in the PC-3 PIP model was up to 1.8-fold higher than the corresponding treatments in a C4-2 model (Table 1) [16].

The mice had a 5–10% body weight decrease in all groups (Figure S7). Weights of the kidneys, liver, and spleen did not reveal any abnormalities in the treatment groups compared to the control group (*p* > 0.05; Figure S8). Hematological analysis showed no difference in white blood cell, red blood cell, or hemoglobin values in the treated mice (*p* > 0.05; Figure S9). Furthermore, levels of serum glutamic oxaloacetic transaminase, glutamic pyruvic transaminase, alkaline phosphatase, and bilirubin indicated no signs of liver toxicity, while creatinine levels suggested no renal toxicity (*p* > 0.05; Figure S10). A reduction in platelet counts were observed in the 2 × 0.2 MBq group (*p* < 0.05 vs. control; Figure S9) and a lower urea level was detected in the 2 × 0.4 MBq group (*p* < 0.05 vs. control; Figure S10), but the parameters were still within the reference range.

### 3.4. PSMA Expression in Xenograft Tumors and Kidneys

**Figure 5:**
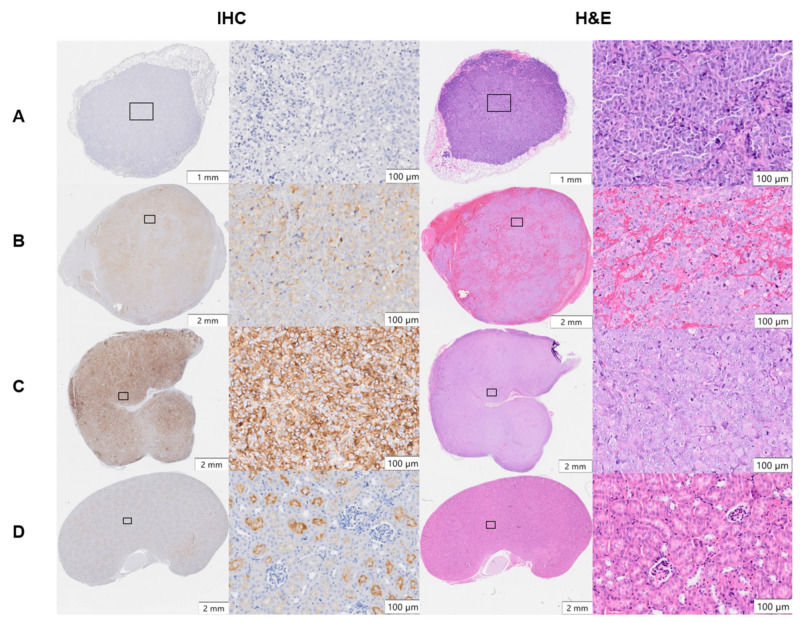
Immunohistochemical (IHC) PSMA staining and hematoxylin and eosin (H&E) staining of (**A**) PC-3 Flu, (**B**) C4-2, and (**C**) PC-3 PIP tumor xenografts and (**D**) kidneys of non-treated mice. Representative histological images (×20 magnification) were taken by using an automatic slide scanner (VS200, Olympus) and analyzed with VS200 ASW software.

PSMA expression in PC-3 Flu, C4-2, and PC-3 PIP xenografts and kidneys was evaluated by IHC (Figure 5). Heterogeneous PSMA expression was detected at a moderate level in C4-2 tumors and at a high level in PC-3 PIP tumors, whereas it was absent in PC-3 Flu tumors. In healthy kidneys, PSMA was exclusively expressed in the luminal parts of the proximal renal tubule’s epithelium and absent in glomeruli and stroma. The isotype control showed no staining in PC-3 PIP tumor and kidney tissues (Figure S11). H&E staining of the xenografts showed blood-rich stroma in C4-2 tumors. However, this was not observed in PC-3 Flu and PC-PIP tumors. (Figure 5).

## 4. Discussion

In the present study, in vitro cytotoxicity and in vivo therapeutic efficacy of the alpha-emitting PSMA radioligand ^212^Pb-NG001 were investigated in two prostate cancer cell lines expressing different levels of PSMA. In addition, the therapeutic effect of repeated administration of the radioligand was investigated.

In vitro cytotoxicity and in vivo therapeutic efficacy of ^212^Pb-NG001 was less than 1.8-fold better for the PC-3 PIP model than for the C4-2 model (Figure 1 and Figure 4 and Table 1), even though PSMA expression is 10–20-fold higher on the surface of PC-3 PIP cells than on C4-2 cells [35]. This discrepancy between the cellular PSMA expression and therapeutic efficacy can be explained by factors including internalization of the radioligand and tumor microenvironment.

The ^212^Pb-NG001 displayed similar binding and internalization ratios as reported for ^44^Sc-, ^152^Tb-, ^212^Pb- and ^177^Lu-labelled PSMA-617 [23,25,62,63]. The genetically engineered PSMA of PC-3 PIP cells do not exhibit the internalizing characteristics of natural PSMA-expressing cells, corresponding to results presented by Tschan et al. [25]. The degree of cellular internalization of alpha-emitting radioligands is an important parameter predicting the effectiveness of TAT because proximity to the nucleus increases the possibility of alpha particle interaction with DNA and double-strand breaks that lead to cell death [64,65,66]. For 50% cell killing, a mean of ~67 and 6 ^212^Pb atoms were bound to the surface of PC-3 PIP and C4-2 cells, respectively, whereas ~4 and 5 ^212^Pb atoms were internalized per cell (Figure 1D,E), indicating the importance of internalization. This is in line with research by Nikula et al. and Azure et al. which reported that only 1 and 11 internalized alpha particles (from ^213^Bi-CHX-A-DTPA-HuM195m and ^212^Pb(PDC)_2_, respectively) were needed to decrease survival to 37–50% [67,68]. Internalization is especially important for the retention of radionuclides with alpha-emitting progenies, such as ^212^Pb, ^225^Ac, and ^227^Th, that could otherwise dissociate from the chelator and possibly translocate to healthy tissues [69]. In the current study, the ratio of total cell-bound ^212^Bi to ^212^Pb activity increased up to 24 h, both for PC-3 PIP and C4-2 cells, suggesting that ^212^Pb-NG001 is internalized and the progeny is retained in the cell (Figure S1).

Our results suggest that 8–15% of alpha particles from cell surface-bound ^212^Pb-NG001 may be responsible for cell death (Figure 1D,E). Alpha particles that are not directly hitting the cell nucleus but still are bound to the cell surface may contribute to the cytotoxic response of the cell population via the bystander effect, in which unirradiated cells exhibit cytotoxic effects due to signals from nearby irradiated cells and via membrane damage. In addition, cells can be crossfire irradiated by alpha particles originating from a neighboring cell [70,71,72]. However, these effects are less prominent for the single cells in the clonogenic assay than in multicellular spheroid models that resemble micrometastases and small avascular tumors [73]. Activity concentrations of only 5–10 kBq/mL of ^212^Pb-NG001 are needed for growth inhibition in C4-2 spheroids [16], whereas 30 kBq/mL is needed for a 50% decrease in clonogenic survival of C4-2 cells (Figure 1C and Table S2). This corresponds to 25–50 MBq and 150 MBq per patient (~5 L blood), respectively. Clinical studies have demonstrated that 50 MBq/patient of ^212^Pb-TCMC-trastuzumab and 175 MBq/patient of ^212^Pb-DOTAMTATE (up to 4 cycles) were well tolerated [74,75]. Therefore, the findings herein indicate that ^212^Pb-NG001 can induce cytotoxic effects in circulating single cancer cells and micrometastases at clinically relevant doses.

The tumor uptake and absorbed dose of ^212^Pb-NG001 in the PC-3 PIP model was up to 1.7-fold higher than in the previously studied C4-2 xenograft model (Figure 2 and Figure S4 and Table S3) [16]. This difference was unexpectedly modest because the higher PSMA level has been previously reported to result in a significantly higher tumor uptake of ^177^Lu-PSMA-617 (6–10-fold higher uptake in PC-3 PIP compared to LNCaP tumors; [25]). However, PC-3 PIP cells were inoculated to mice in medium in the study by Tschan et al. [25], whereas PC-3 PIP cells were inoculated in medium:Matrigel matrix (1:1) in the present study (like for C4-2 cells; [16]), resulting in a very rapid PC-3 PIP tumor growth (Figure S12). This may influence tumor stroma and vascular density, and, thus, radioligand uptake because vascular density tends to decrease with tumor growth [41,42]. Ngen et al. found that higher vascular density enhanced delivery of their PSMA-targeted nanoparticles in the PC-3 PIP tumor peripheries compared to reduced delivery in the low vascularized areas in the centers of the tumor [41]. In the present study, a less blood-rich stroma was observed throughout the whole cross-section of the PC-3 PIP tumor (Figure 5). Several studies report that larger tumors and limited tumor vasculature may result in significant increases in hypoxia burden and tumor necrosis [38,76,77,78,79,80]. The effective molar activity may influence the biodistribution of radioligands by leading to variable degrees of receptor saturation in PSMA-expressing tissues [25,43,44]. In the present study, a more than 5-fold decrease in the effective molar activity of ^212^Pb-NG001 did not have any effect on tumor and kidney uptake (Figure S5), suggesting that the binding sites were not saturated at the tested ligand amounts. This is in line with the previously studied radioligand ^212^Pb-DOTAMTATE by Stallons et al. [48]. In the present study, mice bearing PC-3 PIP tumors treated with 0.2, 0.4, and 0.8 MBq of ^212^Pb-NG001 resulted in TIs of 2.7, 3.0, and 3.5 (effective molar activities of 1.1–1.3 MBq/nmol), respectively (Table 1). In contrast, the PSMA radioligand ^212^Pb-L2 improved TI from 1.9 to 3.0 at much higher activity doses of 1.5 and 3.7 MBq (effective molar activities of 0.7–1.9 MBq/nmol) in a PC-3 PIP model [17]. This may be a result of improved tumor targeting and retention of ^212^Pb-NG001, with 1.4-, 2.8-, and 1.6-fold higher PC-3 PIP tumor uptakes at 1, 4, and 24 h post injection (Figure 2) [17]. The therapeutic efficacy of PC-3 PIP tumor-bearing mice was improved through multiple injections (Figure 4 and Table 1), which may indicate a substantial benefit in therapeutic response from repeated injections of TAT. Three days between injections was chosen because all radioactivity was considered decayed or cleared from the tumor, as the physical half-life of ^212^Pb is 10.6 h. A dosing interval of 14 days was also tested, as this was the time point when tumors started to regrow after injection of the first dose (Figure 4D,E). Because the survival was similar (*p* > 0.05; Figure S6), regardless of the time of the second injection, these data were merged and presented as one group (Figure 4F,G).

Studies with the alpha-emitting radioimmunoconjugates ^225^Ac-lintuzumab, ^211^At-trastuzumab, and ^227^Th-DOTA-p-benzyl-trastuzumab demonstrated no significant improvement in therapeutic efficacy with multiple injections, although reduced radiotoxicity to normal tissues was reported [51,52,81]. The lack of additional therapeutic effects may be explained by the limited tumor penetration of large radioimmunoconjugates and the short range of alpha particles [51]. Conversely, the therapeutic benefits of using multiple injections of the smaller radioligands ^212^Pb-DOTAMTATE, ^225^Ac-L1, and ^149^Tb-PSMA-617 have been demonstrated [22,48,82], and potentially explained by the intratumoral penetration and the possibility of increasing the cumulative dose to the tumor by extending the treatment duration, which allows for systemic toxicity recovery. This preclinical study suggests that a clinically relevant dose of ^212^Pb-NG001 can be estimated to range from 36 to 72 MBq (0.2–0.4 MBq per 30 g mouse; [83]) and from 27 to 51 nmol of NG001 (0.15–0.28 nmol per 30 g mouse) with effective molar activities of 1.1–1.4 MBq/nmol. As mentioned above, this activity range has shown to be well tolerated for clinically used ^212^Pb-TCMC-trastuzumab and ^212^Pb-DOTAMTATE [74,75]. In addition, the ligand dose is expected to be safe as it is low compared to patient dosing (100–200 µg, i.e., 67–133 mmol of PSMA-617; [7,24,84,85]).

**Figure 6:**
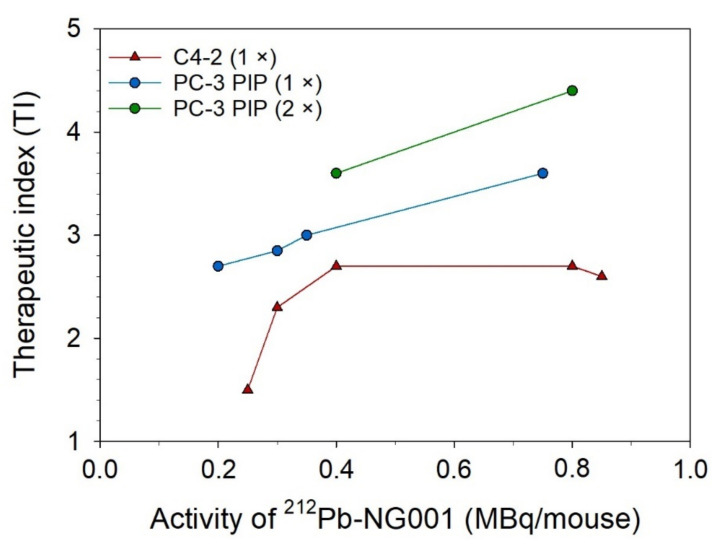
Therapeutic index (TI) of a single dose (1×) or two doses (2×) of ^212^Pb-NG001 (MBq/mouse) in athymic nude mice bearing C4-2 or PC-3 PIP xenografts.

The increased TI of ^212^Pb-NG001 in mice bearing PC-3 PIP tumors compared to C4-2 tumors (Figure 6; [16]) was modest considering the 10-fold higher PSMA expression, which may be explained by the reduced cellular internalization and blood supply to the PC-3 PIP tumors (Figure 1 and Figure 5). The PC-3 PIP tumor model expresses PSMA at a higher level and more homogeneously throughout the xenografts than the C4-2 model (Figure 5), which may not reflect the natural abundance and heterogeneity of PSMA in human cancer [22,35,86]. Thus, the C4-2 tumor model may be more suitable to predict therapeutic efficacy in solid tumors and micrometastases. On the other hand, the reduced blood supply in PC-3 PIP tumors makes them relevant models for nonvascularized metastases.

Selecting appropriate tumor and mouse models can enhance the reproducibility, reliability, and clinical translation of radiopharmaceuticals [25,87]. The most commonly used xenograft models typically involve immunodeficient athymic nude, NOD-SCID, NSG, and BALB-SCID mice with low irradiation tolerance. Previous studies show that greater stromal sensitivity in SCID mice with a resulting reduction in functional tumor vasculature significantly improves tumor growth delay (27% longer in NSG vs. nude mice) and increases tumor response to radiation [54,55]. However, PC-3 PIP tumors showed a lesser extent of blood vessels in our studies (Figure 5), which may be why no difference in treatment response was observed in NSG and athymic nude mice (Figure 3).

## 5. Conclusions

In conclusion, the PSMA-targeting radioligand ^212^Pb-NG001 has a high therapeutic efficacy in prostate cancer models with different PSMA expressions. The PC-3 PIP model displayed a 10-fold higher PSMA expression and radioligand binding than the C4-2 model. Regardless, only a modest increase in the therapeutic efficacy of ^212^Pb-NG001 in the PC-3 PIP model was observed (1.8-fold higher). This may be explained by the lower cellular internalization and less blood-rich stroma of PC-3 PIP xenografts. Repeated administrations of the radioligand significantly improved treatment outcomes in the PC-3 PIP model. Further, these findings indicate that ^212^Pb-NG001 induce therapeutic effects at clinically relevant doses both in C4-2 models that resemble solid tumors and micrometastases with natural PSMA expression and in PC-3 PIP models that better mimic poor vascularized metastases.

## Figures and Tables

**Table 1:** Median survival and therapeutic index (TI) of ^212^Pb-NG001 in athymic nude mice with PC-3 PIP xenografts. The TI was calculated from the median survival of the treated group divided by the median survival of the control group. Statistical significance was estimated by a log-rank test with multiple pairwise comparisons (Holm–Sidak).

Tumor Model	Treatment Group	Effective Molar Activity (MBq/nmol)	Number of Mice	Median Survival	TI	*p*-Value Towards Control
PC-3 PIP	Control		9	10	1	
	0.2 MBq	1.13	5	27	2.7	0.006
	0.4 MBq	1.26	8	30	3.0	0.002
	0.8 MBq	1.24	5	35	3.5	0.006
	2 × 0.2 MBq	1.02	5	36	3.6	0.005
	2 × 0.4 MBq	1.33	12	44	4.4	<0.001
C4-2	Control 1 *		7	23		
	0.25 MBq *	0.5	8	35	1.5	<0.002
	Control 2 *		8	16.5		
	0.3 MBq *	1.1	8	38.5	2.3	0.012
	Control 3 *		6	27		
	0.4 MBq *	2.3	6	74	2.7	<0.001
	0.8 MBq	2.4	6	72.5	2.7	<0.001
	Control 4		7	35		
	0.85 MBq	2.1	6	89.5	2.6	<0.001

* The data is reproduced from [16].

## Data Availability

The data presented in the study are available in the Supplementary Materials or on request from the corresponding author.

## Supplementary Materials

The following supporting information can be downloaded at: https://www.mdpi.com/article/10.3390/cancers14112784/s1. Table S1: Clonogenic survival response of PC-3 Flu, C4-2 and PC-3 PIP cells treated with X-ray irradiation; Table S2: Clonogenic survival response of PC-3 Flu, C4-2 and PC-3 PIP cells treated with 212Pb-NG001; Table S3: Percentage of injected activity per gram of tissue (%ID/g ± SD) of 212Pb-NG001 in athymic nude mice with PC-3 PIP or C4-2 xenografts; Table S4: Statistical significance between PC-3 PIP xenograft groups treated with a single dose or two doses of 212Pb-NG001; Figure S1: Retention of specific cell bound (% of total added activity) 212Pb- and 212Bi-labelled NG001 in C4-2 and PC-3 PIP cells after 1, 2, 4 and 24 h; Figure S2: Survival fraction of C4-2 and PC-3 PIP cells after treatment with X-ray irradiation; Figure S3: Total cell bound and internalized activity of 212Pb-NG001 (mBq/cell) in PC-3 Flu, C4-2 and PC-3 PIP cells; Figure S4: Uptake of 212Pb-NG001 in tumor of athymic nude mice bearing C4-2 or PC-3 PIP xenografts (Bq/g over time); Figure S5: Tumor and kidney uptake of 212Pb-NG001 in nude mice bearing PC-3 PIP or C4-2 xenografts; Figure S6: Survival analysis of PC-3 PIP bearing nude mice treated with saline or 2 × 0.4 MBq of 212Pb-NG001 with dosing interval of 3 or 14 days; Figure S7: Change in body weight of athymic nude mice bearing PC-3 PIP tumors treated with saline or 212Pb-NG001; Figure S8: Relative weight of kidneys, liver and spleen of athymic nude mice with human prostate PC-3 PIP xenografts treated with saline or 212Pb-NG001; Figure S9: Hematological analysis of athymic nude mice with human prostate PC-3 PIP xenografts treated with saline or 212Pb-NG001; Figure S10: Serum parameters of athymic nude mice with human PC-3 PIP xenografts treated with saline or varying doses of 212Pb-NG001; Figure S11: Immunohistochemistry of PC-3 PIP tumor xenografts and kidneys of athymic nude mice using an isotype control antibody; Figure S12: Tumor growth of human prostate PC-3 PIP tumors, with or without Matrigel, in athymic nude mice.
