# Supplementary material for: Factors Influencing the Therapeutic Efficacy of the PSMA Targeting Radioligand 212Pb-NG001
Source: Cancers (Basel). 2022 Jun 3;14(11):2784. doi: 10.3390/cancers14112784 (PMC9179904; doi:10.3390/cancers14112784)
Supplement: Supplementary file 1 [file cancers-14-02784-s001.zip › cancers-1671722-supplementary.pdf]

cancers

MDPI

Supplementary Materials

Factors Influencing the Therapeutic Efficacy of the PSMA Targeting Radioligand ^{212}Pb-NG001

Vilde Yuli Stenberg [1,2,3,*,†], Anna Julie Kjøl Tornes [1,2,3,†], Hogne Røed Nilsen [4], Mona-Elisabeth Revheim [3,5], Øyvind Sverre Bruland [3,6], Roy Hartvig Larsen [2] and Asta Juzeniene [1]

[1] Department of Radiation Biology, Institute for Cancer Research, The Norwegian Radium Hospital, Oslo University Hospital, 0379 Oslo, Norway; anna.julie.kjol.tornes@rr-research.no (A.J.K.T.); asta.juzeniene@rr-research.no (A.J.)
[2] Nucligen AS, 0379 Oslo, Norway; sciencons@gmail.com
[3] Institute of Clinical Medicine, University of Oslo, 0318 Oslo, Norway; monar@ous-hf.no (M.-E.R.); osb@ous-hf.no (Ø.S.B.)
[4] Department of Pathology, Rikshospitalet, Oslo University Hospital, 0372 Oslo, Norway; hogne.roed.nilsen@rr-research.no
[5] Division of Radiology and Nuclear Medicine, Oslo University Hospital, 0379 Oslo, Norway
[6] Department of Oncology, The Norwegian Radium Hospital, Oslo University Hospital, 0379 Oslo, Norway
* Correspondence: vilde.stenberg@rr-research.no; Tel.: +47-9012-8434
† These authors contributed equally to this work.

Table S1. Clonogenic survival response of PC-3 Flu, C4-2 and PC-3 PIP cells treated with X-ray irradiation (mean ± SD, *n* = 3. D_0, dose required to obtain 37% survival; n, number of hits per cell; Alpha, alpha-value; Beta, beta-value and SF2, survival fraction at 2 Gy, were brought out from the survival curves fitted to the multitarget model (equation; $SF = 1 - (1-e^{-D/D0})^n$) and the linear-quadratic model (equation; $SF = e^{-\alpha D-\beta D2}$). SF, survival fraction; D, dose (Gy).

Cell line	D_0 (Gy)	n	Alpha (Gy^{-1})	Beta (Gy^{-2})	SF2
**PC-3 PIP**	1.21 ± 0.07	3.86 ± 1.02	0.33 ± 0.07	0.14 ± 0.04	0.30 ± 0.01
**C4-2**	1.07 ± 0.05	2.67 ± 0.49	0.41 ± 0.04	0.10 ± 0.03	0.29 ± 0.01

Table S2. Clonogenic survival response of PC-3 Flu, C4-2 and PC-3 PIP cells treated with ^{212}Pb-NG001 (mean ± SD, *n* = 3). A37, activity required to obtain 37% survival; A50, activity required to obtain 50% survival. A37 and A50 were brought out from the survival curve (Figure 1C) fitted to the single hit model (equation; $SF = e^{-A/A_0}$).

Cell line	A37 (kBq/mL)	A50 (kBq/mL)
**PC-3 Flu**	242 ± 27	215 ± 61
**C4-2**	43 ± 2	29 ± 4
**PC-3 PIP**	22 ± 1	15 ± 1

Table S3. Percentage of injected activity per gram of tissue (%ID/g ± SD) of ^{212}Pb-NG001 in athymic nude mice with PC-3 PIP (*n* = 5–15) or C4-2 xenografts (*n* = 3–11). The data for PC-3 PIP xenografts is from the current study, while the data for C4-2 xenografts is from [1]. *n*, number of mice.

**Organ**	**Time point**	**Xenograft model**				***p*-value**
		**PC-3 PIP**		**C4-2**		
		%ID/g ± SD	*n*	%ID/g ± SD	*n*	
Tumor	1 h	30.7 ± 6.4	5	23.3 ± 8.7	3	0.209
	2 h	20.5 ± 5.2	15	17.6 ± 6.8	11	0.224
	4 h	23.2 ± 5.7	5	13.6 ± 2.1	5	0.007
	24 h	13.2 ± 1.7	5	11.3 ± 3.4	3	0.346
Kidneys	1 h	50.4 ± 27.8	5	62.1 ± 7.0	3	0.517
	2 h	8.2 ± 3.4	15	21.1 ± 10.3	11	0.001
	4 h	7.0 ± 1.1	5	9.6 ± 2.5	5	0.061
	24 h	3.2 ± 0.6	5	5.2 ± 0.8	3	0.095

Table S4. Statistical significance between PC-3 PIP xenograft groups treated with a single dose or two doses of ^{212}Pb-NG001. Athymic nude mice received a single dose of 0.2 MBq (*n* = 5), 0.4 MBq (*n* = 8) or 0.8 MBq (*n* = 5), or two doses of 0.2 MBq (*n* = 5) or 0.4 MBq (*n* = 12) ^{212}Pb-NG001. Statistical significance was estimated by a log-rank test with Holm-Sidak pairwise comparison. *n*, number of mice.

**Treatment group comparisons**	**P-value (single vs. double dose)**
1 × 0.2 MBq vs. 2 × 0.2 MBq	0.031
1 × 0.4 MBq vs. 2 × 0.2 MBq	0.04
1 × 0.4 MBq vs. 2 × 0.4 MBq	< 0.001
1 × 0.8 MBq vs. 2 × 0.4 MBq	0.016

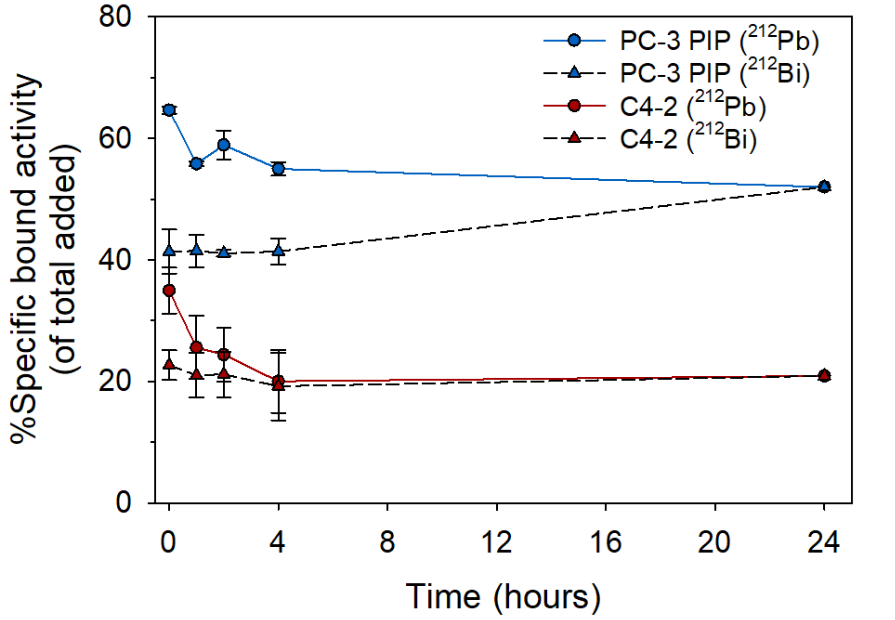

Figure S1. Retention of specific cell bound (% of total added activity) ^{212}Pb- and ^{212}Bi-labelled NG001 in C4-2 and PC-3 PIP cells after 1, 2, 4 and 24 hours, n = 2–3. The C4-2 and PC-3 PIP cells were incubated with 30 kBq/mL and 15 kBq/mL of ^{212}Pb-NG001, respectively, which was the activity reducing survival with 50% (Figure 1C).

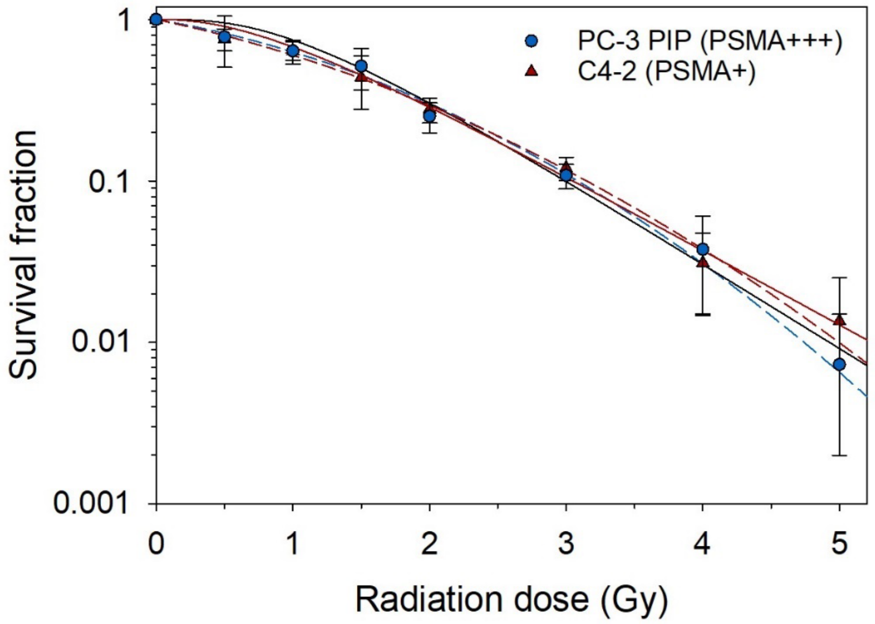

Figure S2. Survival fraction of C4-2 and PC-3 PIP cells after treatment with X-ray irradiation (mean ± SD, n = 3). The survival curves were fitted to the multitarget model (solid line, equation; $SF = 1 - (1-e^{-D/D0})^n$) and the linear-quadratic (dashed line, equation; $SF = e^{-\alpha D-\beta D2}$) model. SF, survival fraction; D, dose (Gy); D_0, dose required to obtain 37% survival; n, number of hits per cell.

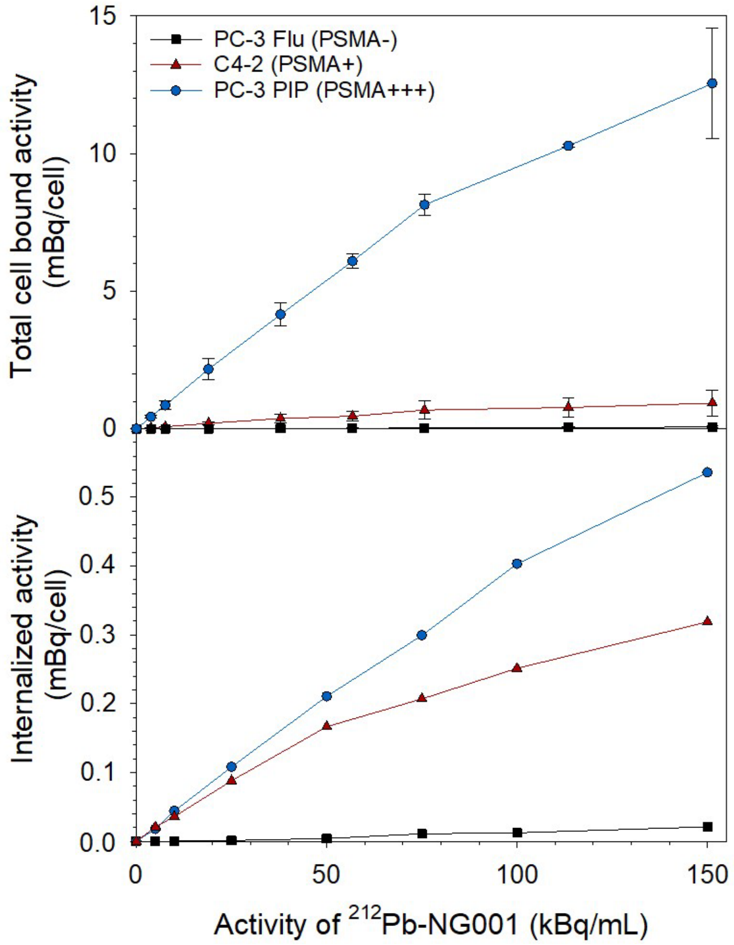

Figure S3. Total cell bound (upper) and internalized (lower) activity of ^{212}Pb-NG001 (mBq/cell) in PC-3 Flu, C4-2 and PC-3 PIP cells, *n* = 3.

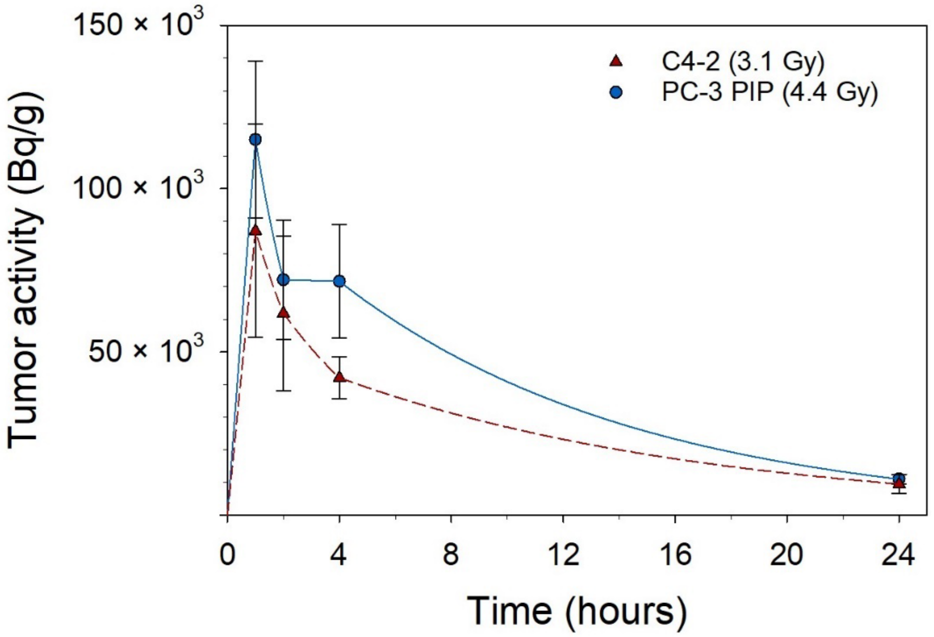

Figure S4. Uptake of ^{212}Pb-NG001 in tumor of athymic nude mice bearing C4-2 or PC-3 PIP xenografts (Bq/g over time), n = 3–15 mice per time point.

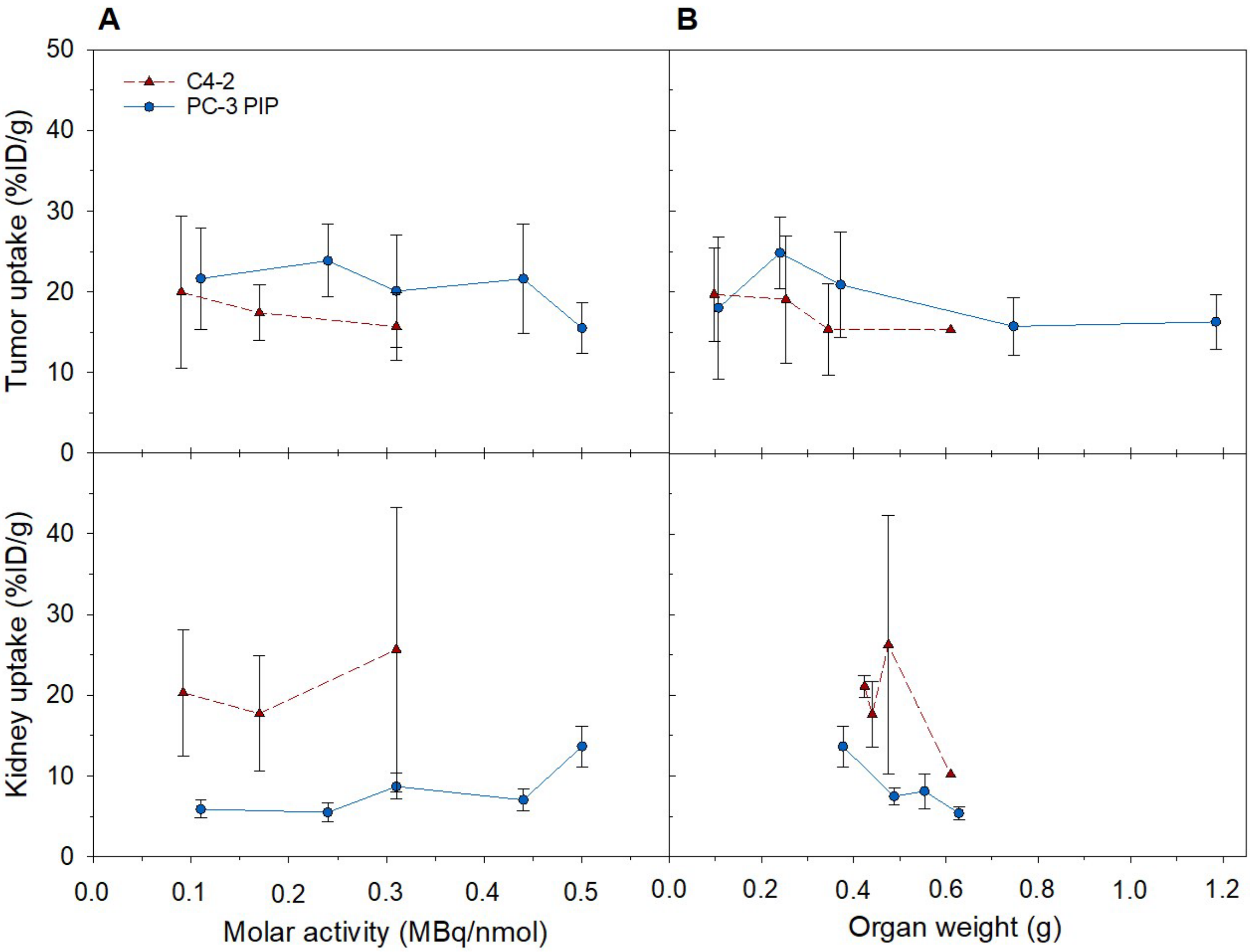

Figure S5. Tumor and kidney uptake of ^{212}Pb-NG001 in nude mice bearing PC-3 PIP or C4-2 xenografts. The uptakes are shown at (**A**) different effective molar activities (MBq/nmol NG001) and (**B**) organ weights at 2 hours post injection

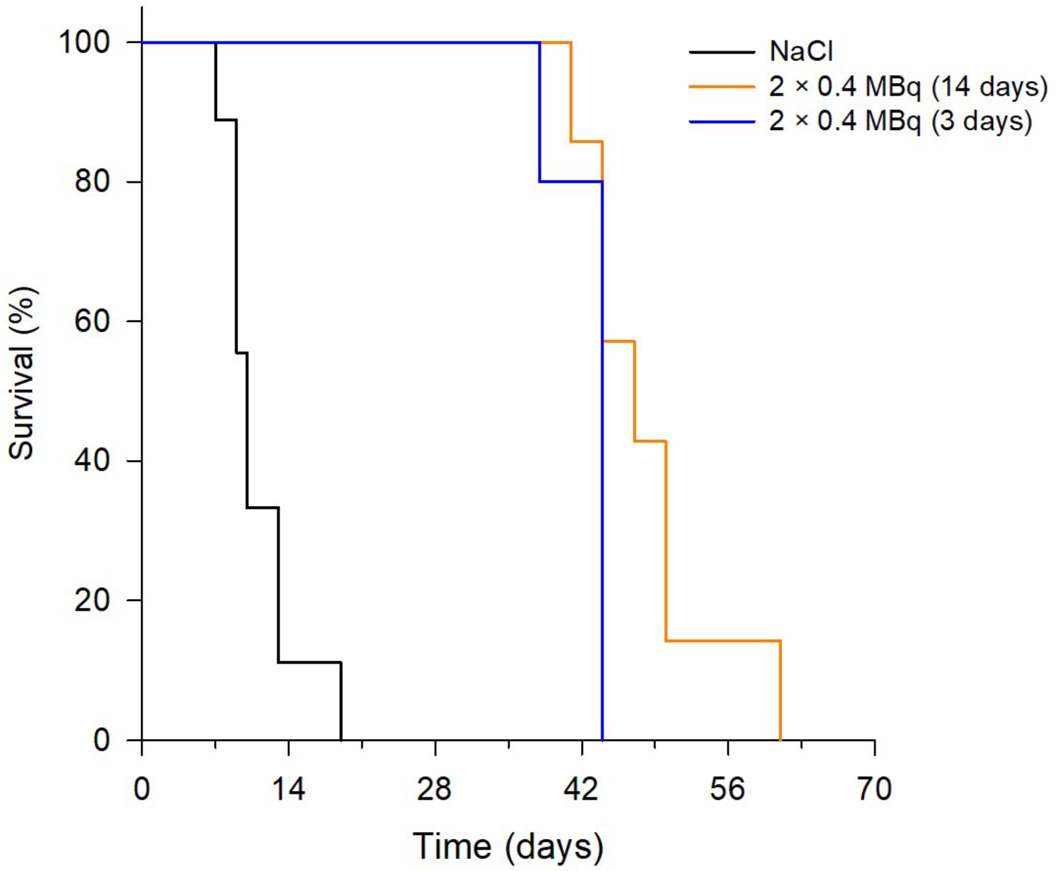

Figure S6. Survival analysis of PC-3 PIP bearing nude mice treated with saline ($n = 9$) or 2 × 0.4 MBq of ^{212}Pb-NG001 with dosing interval of 3 ($n = 5$) or 14 days ($n = 7$). Survival curves were estimated by the Kaplan-Meier method and the curves of the different groups were compared using a log-rank test with multiple pairwise comparisons (Holm-Sidak). $p > 0.05$.

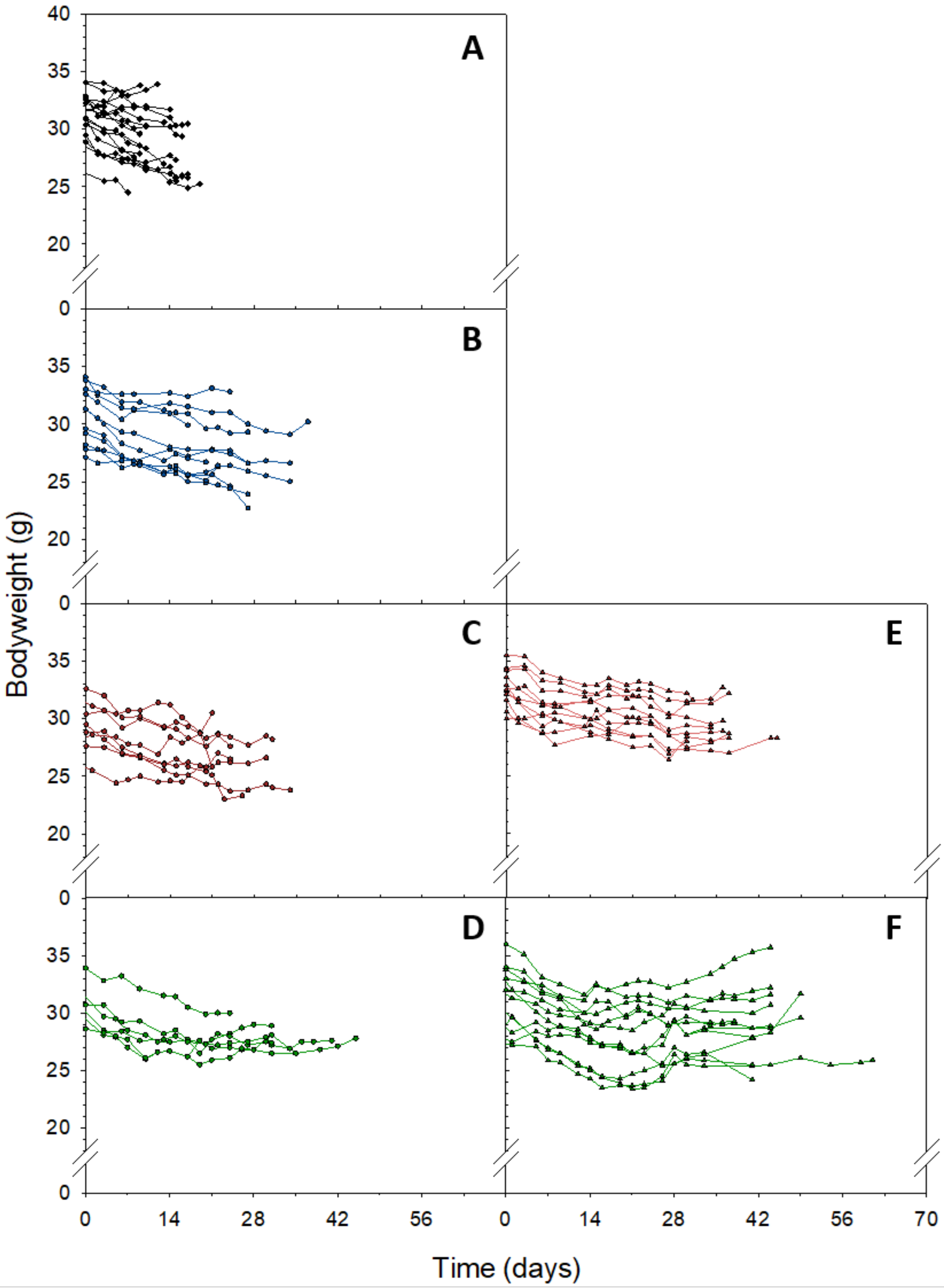

Figure S7. Change in body weight of athymic nude mice bearing PC-3 PIP tumors treated with saline or ^{212}Pb-NG001. The treatment groups received (**A**) saline (*n* = 9), (**B**) 0.2 (*n* = 5), (**C**) 0.4 (*n* = 8), (**D**) 0.8 (*n* = 5), (**E**) 2 × 0.2 (*n* = 5), or (**F**) 2 × 0.4 (*n* = 12) MBq of ^{212}Pb-NG001.

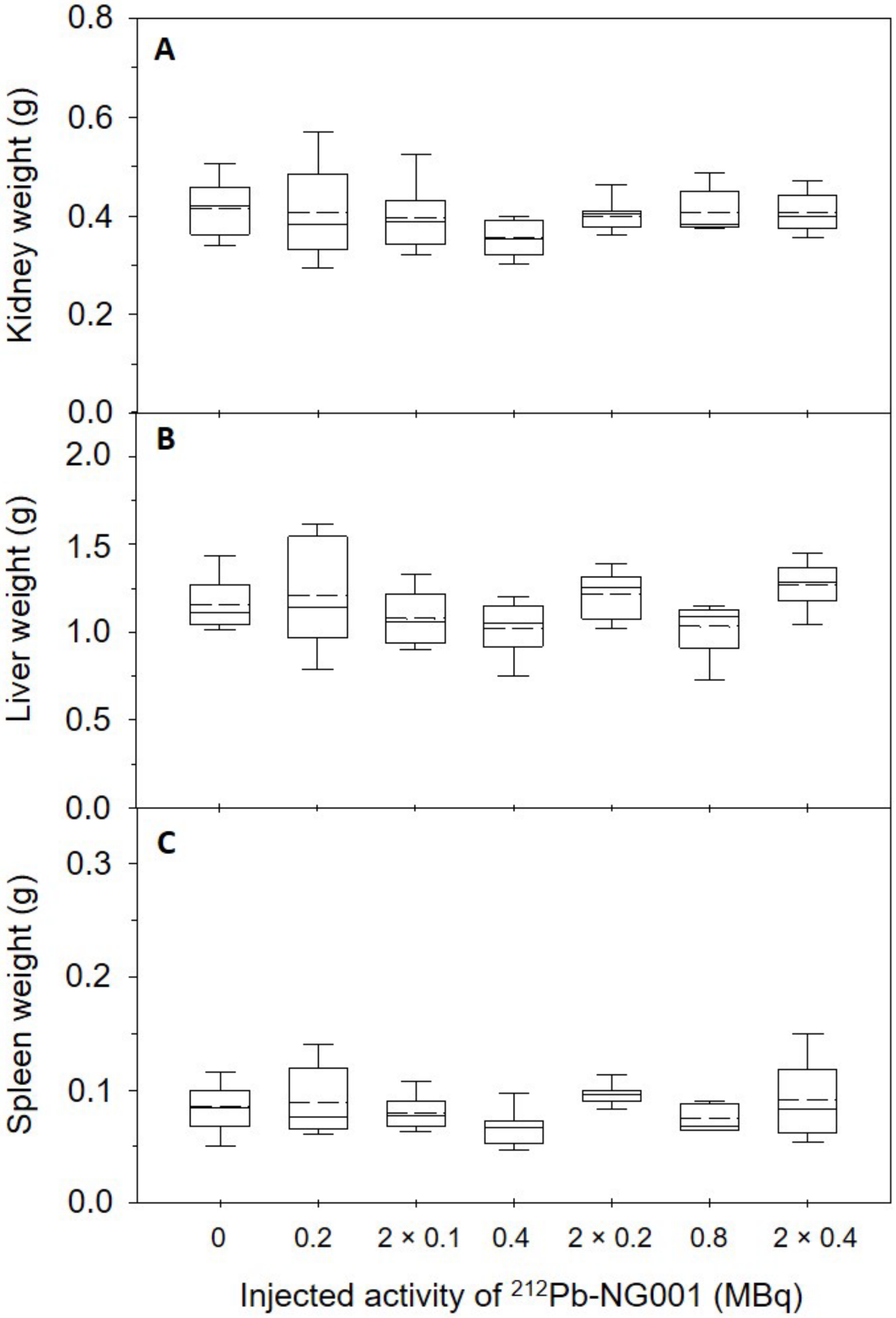

Figure S8. Relative weight (%organ weight of body weight) of (**A**) kidneys, (**B**) liver and (**C**) spleen of athymic nude mice with human prostate PC-3 PIP xenografts treated with saline or ^{212}Pb-NG001. The mice were administered saline (n = 9), 0.2 MBq (n = 5), 0.4 MBq (n = 8), 0.8 MBq (n = 5), 2 × 0.2 MBq (n = 5) or 2 × 0.4 MBq (n = 12) of ^{212}Pb-NG001. Bottom of the boxes represent the 25th percentiles, dashed lines present the means, solid lines represent the medians, top of the boxes represent the 75th percentiles and whiskers represent the 5th and 95th percentiles.

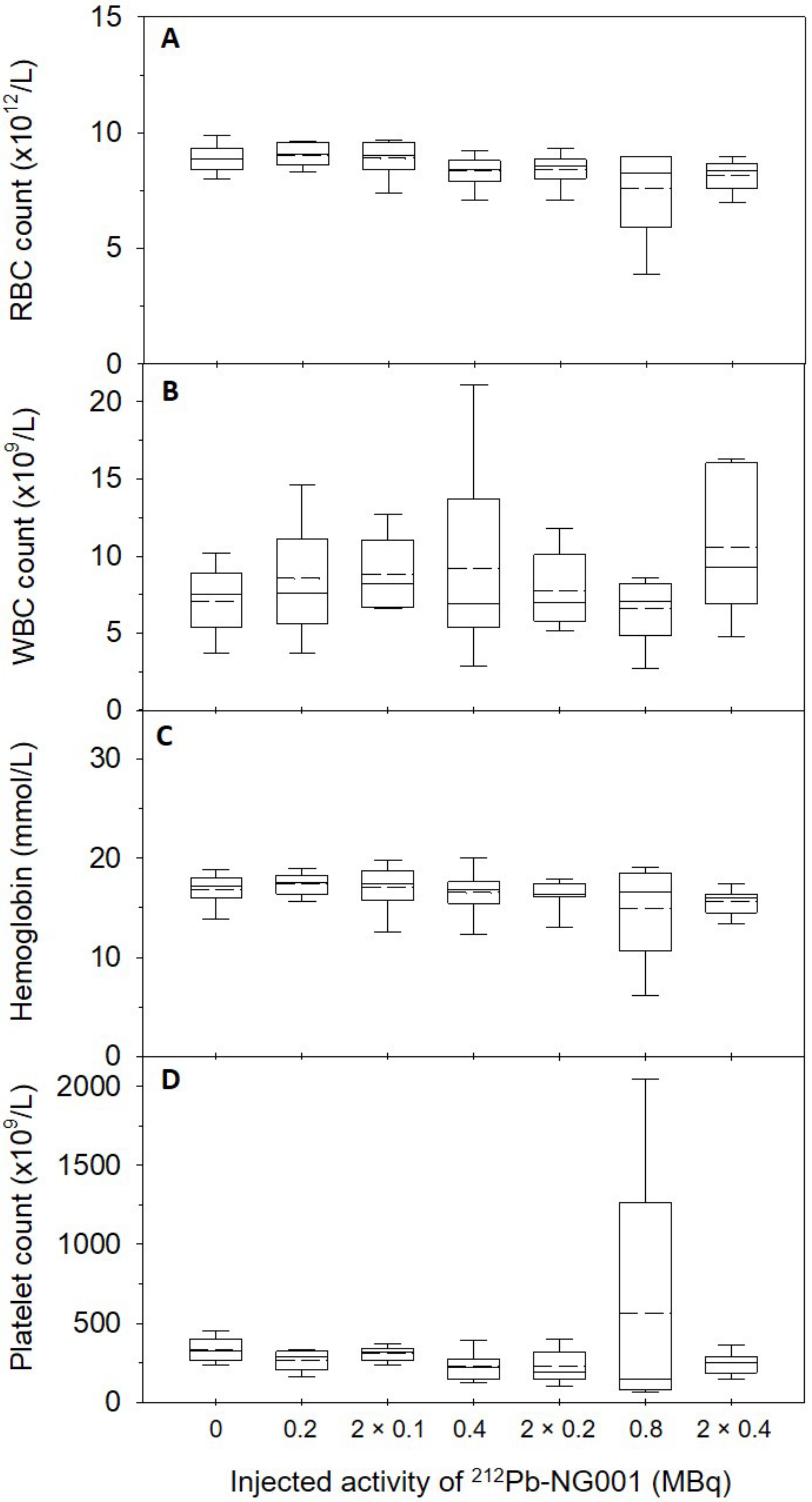

Figure S9. Hematological analysis of athymic nude mice with human prostate PC-3 PIP xenografts treated with saline or ^{212}Pb-NG001. (**A**) Red blood cell (RBC), (**B**) white blood cell (WBC), (**C**) hemoglobin and (**D**) platelet count of athymic nude mice treated with saline (*n*= 9), 0.2 MBq (*n*= 5), 0.4 MBq (*n* = 8), 0.8 MBq (*n*= 5), 2 × 0.2 MBq (*n*= 5) or 2 × 0.4 MBq (*n*= 12) of ^{212}Pb-NG001. *$p < 0.05$.

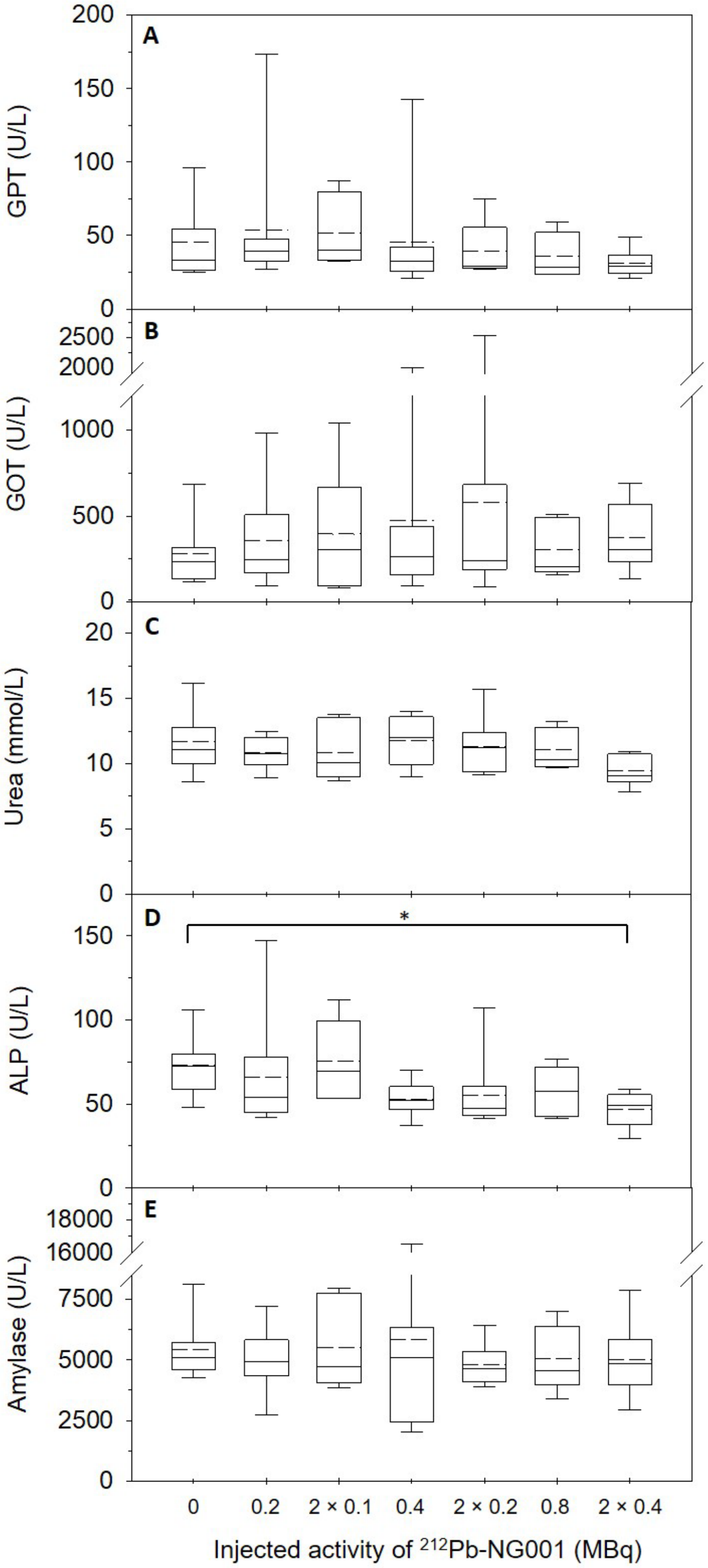

Figure S10. Serum parameters of athymic nude mice with human PC-3 PIP xenografts treated with saline or varying doses of ^{212}Pb-NG001. (**A**) GPT, (**B**) GOT, (**C**) urea, (**D**) ALP and (**E**) amylase values of mice treated with saline (*n* = 9), 0.2 MBq (*n* = 5), 0.4 MBq (*n* = 8), 0.8 MBq (*n* = 5), 2 × 0.2 MBq (*n*= 5) or 2 × 0.4 MBq (*n*= 12) of ^{212}Pb-NG001. Bilirubin < 8.55 µmol/L and creatinine < 44.2 µmol/L was measured for all mice, except for one mouse treated with 2 × 0.2 MBq ^{212}Pb-NG001 with bilirubin of 9.2 µmol/L. GOT, glutamic oxaloacetic transaminase; GPT, glutamic pyruvic transaminase; ALP, alkaline phosphatase. *p < 0.05.

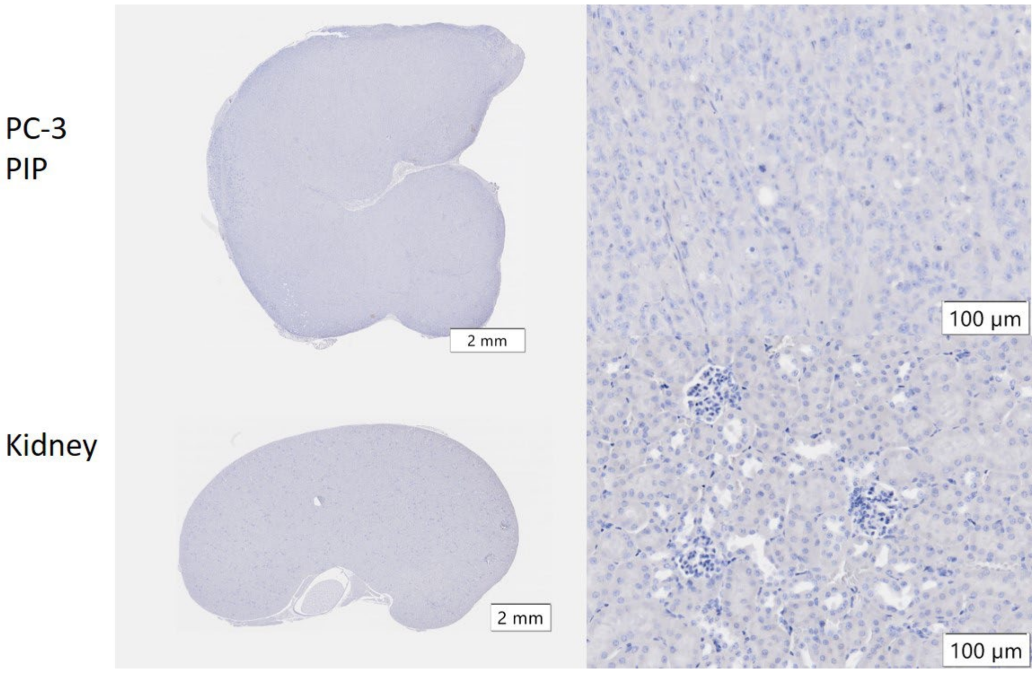

Figure S11. Immunohistochemistry (IHC) of PC-3 PIP tumor xenografts and kidneys of athymic nude mice using an isotype control antibody. Representative histological images (× 20 magnification) were taken using an automatic slide scanner (VS200, Olympus) and analyzed with the VS200 DESKTOP ASW 3.2 software.

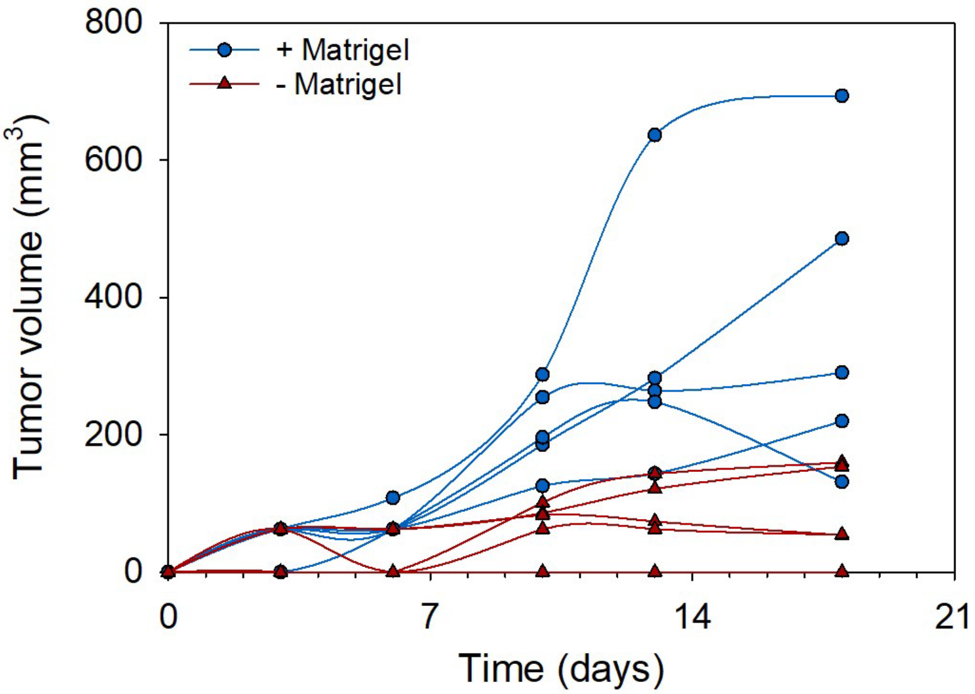

Figure S12. Tumor growth of human prostate PC-3 PIP tumors, with or without Matrigel, in athymic nude mice. Before cell inoculation, the tumor cells were resuspended in fresh medium:Matrigel Matrix (1:1) or in fresh medium only.

References

1. Stenberg, V.Y.; Larsen, R.H.; Ma, L.W.; Peng, Q.; Juzenas, P.; Bruland Ø, S.; Juzeniene, A. Evaluation of the PSMA-Binding Ligand (212)Pb-NG001 in Multicellular Tumour Spheroid and Mouse Models of Prostate Cancer. *Int. J. Mol. Sci.* **2021**, *22*, doi:10.3390/ijms22094815.
